# Intralesional Tetracycline Injection, Pinch Technique, and Canthopexy for the Treatment of Severe Festoons: Preliminary Results

**DOI:** 10.1093/asjof/ojab048

**Published:** 2021-11-20

**Authors:** Sergio Lessa, João Pontello, Deilton Duarte, Diogo Lobão

**Affiliations:** Division of Plastic Surgery, State University of Rio de Janeiro, Rio de Janeiro, Brazil

## Abstract

**Background:**

Many techniques have been presented for the treatment of lower eyelid festoons, but no singular technique has become dominant.

**Objectives:**

The authors describe the safety and efficacy of intralesional tetracycline injection, the pinch technique, and canthopexy for the treatment of severe festoons.

**Methods:**

Institutional board review approval was obtained, and a retrospective chart review was performed on 15 consecutive patients who had received 2% tetracycline injections to treat lower eyelid large festoons between February 2017 and February 2020. Three months after the last injection, a series of patients underwent the surgical procedure: pinch technique and canthopexy bilaterally.

**Results:**

Clinical and photographic records were reviewed, and 12 patients were included in the analysis. Three patients did not return for follow-up after the injection series. Of the 12 patients, there were 3 male patients and 9 female patients, with an average age of 66.6 years. The mean volume injected in each festoon was 0.43 mL, and the mean follow-up was 313 days. A series of injections with a 3-month time interval were performed for patients with a partial response to the initial injection. There was no evidence of complications at the site of the injection. Three months after the last injection, these 12 patients underwent complementary surgical treatment, which included pinch resection and canthopexy.

**Conclusions:**

These preliminary results suggest that intralesional injections of tetracycline 2% may offer a safe option to treat lower eyelid festoons. This noninvasive procedure represents adjunct benefits to complementary surgical therapy.

**Level of Evidence: 4:**

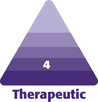

Eyelids and periorbital changes are the first detectable signs of aging. Among the many changes that occur, festoons and malar bags present a challenge to the treating physician. Furnas was the first to describe the anatomic changes of festoons as well as an approach to treating these conditions.^1^ Festoons are caused by laxity and degeneration of the skin and *orbicularis* muscle. Kpodzo et al described festoons as “cascading hammocks of lax skin and orbicularis muscle that hang between the medial and lateral canthi and may or may not contain herniated fat.” ^2^ Festoons are normally located within the prezygomatic space, which is bound by the orbitomalar ligament superiorly and the zygomaticocutaneous ligament inferiorly.^2,3^ Muzaffar et al cited the elongation of the orbitomalar ligament as a key to aging changes of the lower eyelid and the appearance of malar mounds.^4^ Anatomic researchers Pessa and Garza proposed that festoons result from malar edema progression, causing malar mounds and eventually resulting in festoons.^5^ The etiology of festoons is thus multifactorial.

Many different surgical approaches have been attempted with variable success rates. The invasive treatment of malar and festoon bags depends on the diagnosis, the patient’s age, and the severity of the lesions. The treatment options include microsuction,^6-8^ myocutaneous flap-*orbicularis* muscle resection,^1,9,10^ midface lifts,^2,11^ and direct excision of the festoon.^2,12,13^ Nonsurgical treatment options consist of interventions with variable results and may represent adjunct benefits to surgical therapies. Some of these options include Kinesio tape to minimize festoons,^14^ the use of radiofrequency microneedling,^15^ laser management of festoons,^16^ hyaluronidase injection,^17^ and tetracycline-doxycycline injections.^18,19^ Two studies focused on the use of these antimicrobials in the periorbital treatment of festoons and provided new insight into the field. However, the applications of these drugs provide only initial results, thus requiring more detailed studies to determine the safety and efficiency of this treatment.

In 2020, Chon et al evaluated the long-term patient experience with tetracycline injections for treatment of festoons, a retrospective review of 102 patients. In this review, intralesional tetracycline injections appear to improve festoons in the majority of patients with very few side effects.^20^

The purpose of this report is to review the authors’ preliminary experience injecting tetracycline 2% and the associated surgical procedure for the treatment of aesthetically undesirable lower eyelid festoons.

## METHODS

The study was approved by the ethics and research committee (Plataforma Brasil System, approved under number: 3.081.115) and conducted according to ethical standards of the Helsinki Declaration 1964 and its subsequent amendments. A retrospective chart review was performed on 15 consecutive patients who had received 2% tetracycline injections to treat lower eyelid large festoons at the Department of Plastic Surgery of State University of Rio de Janeiro between February 2017 and February 2020. All patients with acquired festoons gave a history of puffiness and bags between the lower eyelids and midface. The deformities began with aging and were not present in youth. Data collection included the following: age, sex, date and volume of injection, number of injections, and subjective complaints. Statistical analysis was performed with the aid of SPSS (IBM Corp. Released 2012. IBM SPSS Statistics for Machintosh, Version 21.0. Armonk, NY). Continuous variables are described as the mean, standard deviation (SD), and minimum and maximum values. Analytical statistics were performed using the Wilcoxon signed-rank test with a significance level when *P* was less than 0.05.

We used the same method as Perry et al to obtain a reconstituted tetracycline 2% solution from 02 g tetracycline HCl USP powder (Fagron, St. Paul, MN), adding sterile water, and filtering to rebuild tetracycline 2% solution.^18^

The solution was injected into the cavity of the festoon in several planes between the *orbicularis* and deep fascia using a 1-mL syringe attached to a 30-gauge needle (Video). All the procedures were performed at the outpatient clinic without sedation.

The volume of injection was guided by the volume of the festoon. Repeat injections were performed in patients as needed for residual volume following an observation period of 3 months. Three months after the last injection, a series of patients underwent the surgical procedure, which included “pinch resection” and muscle suspension canthopexy bilaterally. The pinch technique can be performed under local anesthesia. Using a 7- to 8-mm skin pinch technique with the patient in an upward gaze, the greatest amount of skin is drawn upward for excision to avoid traction on the lower lid margin.

In all cases, the *orbicularis* is suspended by a single suture anchoring the lateral portion of the *pretarsal orbicularis* muscle to the lateral orbital periosteum. When the suture is tied, it pulls the muscle upward and laterally, creating a tight sling under the lower eyelid. Additional elevation is done through another canthopexy, affixing the dermis of the lower eyelid wound to the periosteum, above the lateral angle. A running suture is used for the final closure (Video). All pretreatment and posttreatment photographs were taken in a standardized position and were reviewed by 2 independent physicians.

## RESULTS

A total of 15 patients underwent tetracycline 2% injection for the treatment of large bilateral lower eyelid festoons. Clinical and photographic records were reviewed, and 12 patients were included in the analysis. Three patients were excluded because they had not returned for follow-up after a series of injections were completed. During our phone contacts, these patients expressed satisfaction with the injection results and were not ready to return for further treatment. It is important to mention that patients who are attended in our service arrive by way of a central referral system of the Public Health Service of the State of Rio de Janeiro. Often, they live extremely far making travel, especially in pandemic times, more challenging. Of the 12 included patients, 24 treatment sides were analyzed. After tetracycline 2% injections, there were 3 male patients and 9 female patients with an average age of 66.67 years (range, 64-70 years with a SD of 2.16 years). All patients received bilateral injections at the outpatient clinic without sedation. The mean injected volume of tetracycline in each festoon was 0.43 mL (range, 0.35-0.60 mL and SD 0.91 mL). Patients returned for follow-up 1 week after each injection at 3-month intervals. At each return visit, the reduction of volume and the degree of aesthetic improvement determined subsequent injections. In our cases of very severe festoons, patients underwent a series of either 3 or 4 injections. The mean number of injections performed per site was 3.16 (range, 3-4). The mean follow-up was 313 days (range, 268-380 days and SD of 47.1 days). The tetracycline injection used in this study routinely caused moderate to intense transient pain at the site of injection. A series of injections with a 3-month time interval were performed for patients with a partial response to the initial injection. The 3-month interval between injections was established in accordance with the average period of response to tetracycline.^18-20^ There was no evidence of erythema, induration, numbness, dermatologic complications, or necrosis of the injection site at the 1-week postinjection visit. All 12 patients were extremely satisfied with the results. The only complaint manifested was the burning sensation at the moment of the injection lasting up to 10 minutes, as has been reported in the literature. Clinically, tetracycline seems to improve festoons within 3 months. Three months after the last injection, 12 patients underwent bilateral muscle suspension canthopexy (Table 1).

**Table 1.** Mean Volumes per Site in Each Injection

	First injection		Second injection		Third injection		Forth injection	
	Right	Left	Right	Left	Right	Left	Right	Left
Number of patients	12		12		8		1	
Mean volumes (SD)	0.45 (0.07)	0.45 (0.06)	0.44 (0.07)	0.44 (0.06)	0.39 (0.05)	0.38 (0.05)	0.35^a^	0.30^a^

SD, standard deviation. ^a^Absolute value.

**Figure 1.**
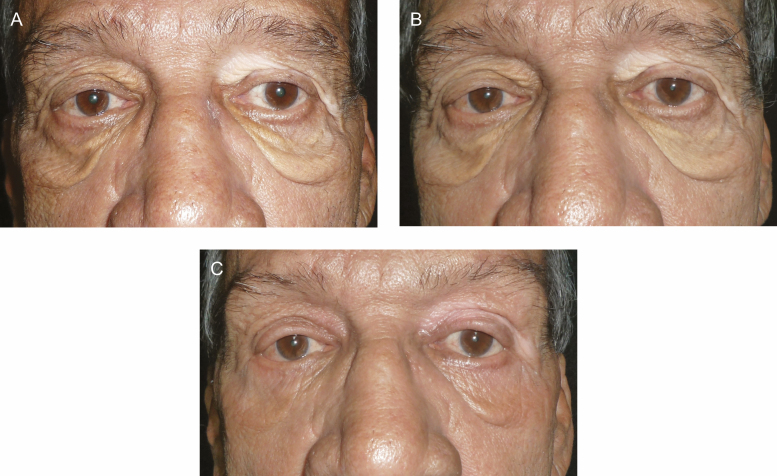
A 68-year-old male with cascading festoons (A) before treatment; (B) showing a decrease in festoon volume after 3 bilateral injections of tetracycline at 9 months; and (C) postsurgery, month 10 of treatment, showing improvement with some residual festoon presence on the left side.

**Figure 2.**
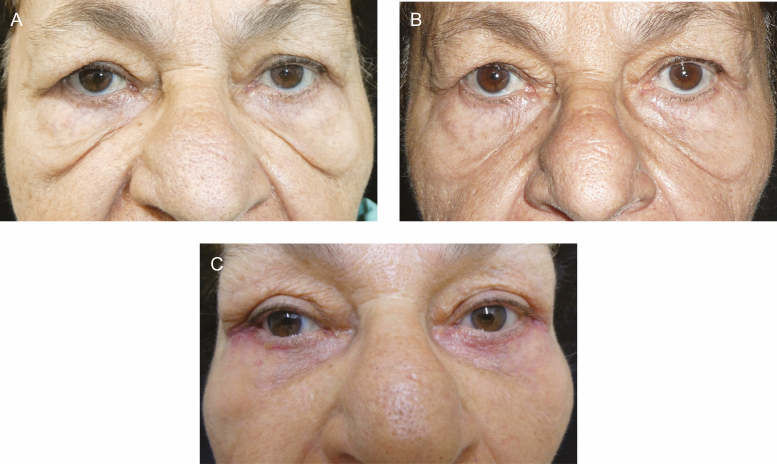
A 70-year-old female with severe bilateral festoons (A) before treatment; (B) showing a decrease in festoon volume after 3 bilateral injections of tetracycline at 9 months; and (C) postsurgery, month 10 of treatment, showing improvement of festoons.

**Figure 3.**
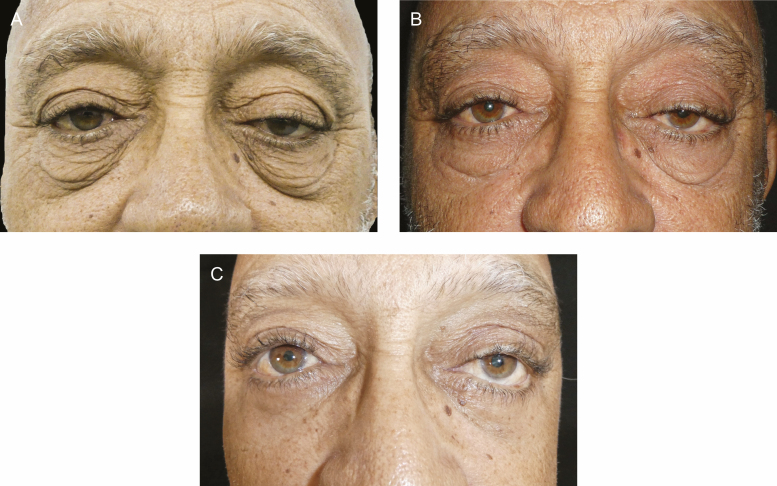
A 65-year-old male with cascading festoons (A) before treatment; (B) showing a decrease in festoon volume after 4 bilateral injections of tetracycline at 9 months; and (C) postsurgery, month 12 of treatment, showing festoon improvement.

**Figure 4.**
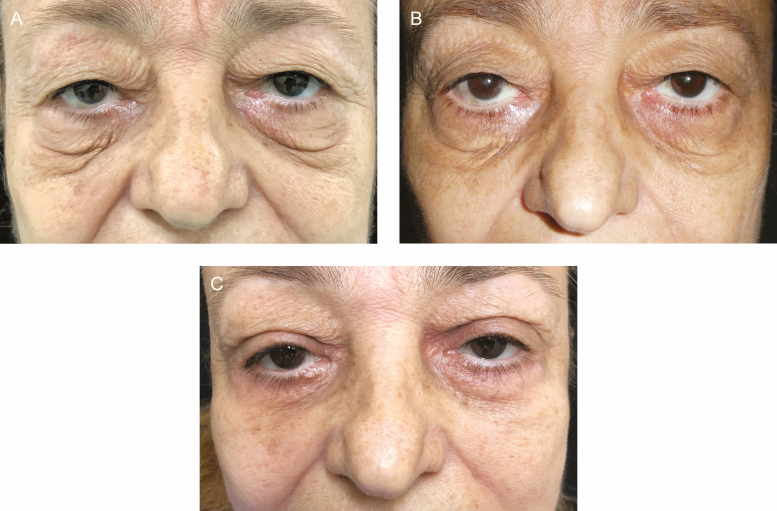
A 63-year-old female with cascading festoons (A) before treatment; (B) showing a decrease in festoon volume after 3 bilateral injections of tetracycline at 9 months; and (C) postsurgery, month 12 of treatment, showing festoon improvement.

The volume differences between the first injection and the second and third injections were analyzed using the Wilcoxon signed-rank test, with a *P-*value lower than 0.05 indicating significance. The differences observed between the first and second injections on the right side resulted in a *P-*value of 0.46 and the first compared with the third had a *P-*value of 0.26. In the left side analysis, a *P*-value of 0.46 was obtained for the comparison between the first and the second sides, and a *P-*value of 0.24 was obtained for the comparison with the third side. These results show that the differences in volume are statistically significant. The final results of the association of the procedures showed a significant cosmetic improvement, although in 2 patients, very discreet unilateral festoon residue remained even after surgery. Both these patients were nonetheless satisfied with the results and did not wish to undergo further surgical correction as was offered to them. Pretreatment and posttreatment photographs are shown in Figures 1-4. 

## DISCUSSION

The tetracycline family consists of compounds with antimicrobial actions. They also exhibit numerous nonantimicrobial properties. For example, they produce growth factor-like activity that stimulates fibroblast proliferation and have also demonstrated the ability to inhibit matrix metalloproteinases from numerous cellular tissues to promote collagen and fibrin deposition.^20,21^ Tetracycline compounds have been used to successfully achieve chemical pleurodesis^21-26^ and to treat a variety of ophthalmic conditions, such as chronic bulbar chemosis.^27^

Three recent studies focused on the use of antimicrobials in the treatment of malar mounds and festoons.^18-20^ In 2 of them, the intralesional injection of tetracycline 2% showed improvement in the contour to correct the festoons.^18,20^ Another antibiotic in the tetracycline family, doxycycline, was used at a concentration of 10 mg/mL to correct festoons and malar edema 19_._

Clinically, tetracycline seems to improve festoons and may achieve a final result several months after injection.^18-20^ The patients included in this study presented grade 3 bilateral festoons, according to the classification proposed by Lam et al.^28^ Three months after the injections of tetracycline 2%, the change in appearance of the festoons was visible due to dense adhesions and fibrosis at the local tissues. Repeated injections were performed for patients with partial response to the initial injections. In our experience, tetracycline injections are an effective treatment of festoons, although sequential injections are needed to produce desired volume change in very severe festoons. It is difficult to distribute a small fraction of tetracycline solution in different planes of the festoons. Tetracycline must be injected into the cavity of the festoon between the deep fascial plane and the *orbicularis* in the subcutaneous and intramuscular planes. The most common adverse effect with tetracycline injections is discomfort during the injection, usually lasting approximately 15-20 minutes. Periorbital nerve block (infraorbital and zygomaticofacial nerves) can relieve patient comfort during the injections.

These preliminary clinical observations encouraged us to submit 12 patients to subsequent treatment with the pinch technique to remove excess skin and canthopexy for final lateral support of the lower eyelid. The association of the procedures may represent an additional treatment modality for improving the appearance of severe lower eyelid festoons. While some residual festoon presence may remain after the combined procedure, the dramatic aesthetic change in the appearance of all patients is visible and satisfactory.

The limitations of this study include its retrospective nature and the reduced number of cases studied. Further studies may determine optimal treatment doses and intervals as well as any possible significant complications.

## CONCLUSIONS

These preliminary results suggest that intralesional injections of tetracycline 2% may offer a safe option to treat acquired, lower eyelid festoons. In our experience, tetracycline injections improve festoon appearance, and the procedure is well accepted by patients. This noninvasive procedure represents adjunct benefits to complementary surgical therapy. The association of these procedures may represent an additional treatment modality to improve the appearance of severe lower eyelid festoons.
